# μ-η^2^:η^2^-Peroxido-bis­[nitratodioxido­bis(pyrrolidin-2-one)uranium(VI)]

**DOI:** 10.1107/S1600536810013449

**Published:** 2010-04-21

**Authors:** Koichiro Takao, Yasuhisa Ikeda

**Affiliations:** a Institute of Radiochemistry, Forschungszentrum Dresden-Rossendorf, PO Box 51 01 19, 01314 Dresden, Germany; b Research Laboratory for Nuclear Reactors, Tokyo Institute of Technology, 2-12-1-N1-34, O-okayama, Meguro-ku, Tokyo 152-8550, Japan

## Abstract

In the crystal structure of the title compound, [U_2_(NO_3_)_2_O_4_(O_2_)(C_4_H_7_NO)_4_], two UO_2_^2+^ ions are connected by a μ-η^2^:η^2^-O_2_ unit. The O_2_ unit shows ‘side-on’ coordination to both U atoms. An inversion center is located at the midpoint of the O—O bond in the O_2_ unit, affording a centrosymmetrically expanded dimeric structure. The U—O(axial) bond lengths are 1.777 (4) Å and 1.784 (4) Å, indicating that the oxidation state of U is exclusively 6+, *i.e.*, UO_2_^2+^. Furthermore, the O—O distance is 1.492 (8) Å, which is typical of peroxide, O_2_^2–^. The U atom is eight-coordinated in a hexa­gonal-bipyramidal geometry. The coordinating atoms of the nitrate and pyrrolidine-2-one ligands and the μ-η^2^:η^2^-O_2_^2–^ unit are located in the equatorial plane and form an irregular hexa­gon. An inter­molecular hydrogen bond is found between N—H of the pyrrolidine-2-one ligand and the coordinating O of the same ligand in a neighboring complex. A second inter­molecular hydrogen bond is found between the N—H of the other pyrrolidine-2-one ligand and one of the uranyl oxido atoms.

## Related literature

For the structural chemistry of uran­yl(VI)–peroxido complexes, see: Haegele & Boeyens (1977 ▶); Charpin *et al.* (1985 ▶); Doyle *et al.* (1993 ▶); Rose *et al.* (1994 ▶); Thuéry *et al.* (1999 ▶); de Aquino *et al.* (2001 ▶); John *et al.* (2004 ▶); Masci & Thuéry (2005 ▶); Zehnder *et al.* (2005 ▶); Kubatko *et al.* (2007 ▶); Ikeda *et al.* (2007 ▶); Takao *et al.* (2009 ▶); Vaska (1976 ▶).
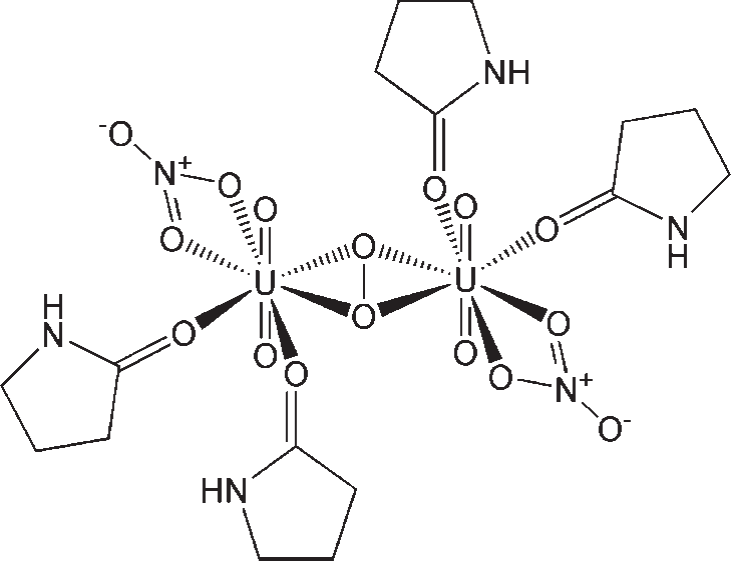


## Experimental

### 

#### Crystal data

[U_2_(NO_3_)_2_O_4_(O_2_)(C_4_H_7_NO)_4_]
*M* *_r_* = 1036.50
Triclinic, 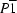

*a* = 8.783 (2) Å
*b* = 8.899 (3) Å
*c* = 9.587 (3) Å
α = 68.24 (3)°
β = 81.30 (2)°
γ = 68.96 (2)°
*V* = 649.4 (3) Å^3^
*Z* = 1
Mo *K*α radiation
μ = 12.54 mm^−1^
*T* = 173 K
0.30 × 0.20 × 0.20 mm

#### Data collection

Rigaku R-AXIS RAPID diffractometer
Absorption correction: numerical (*NUMABS*; Higashi, 1999 ▶) *T*_min_ = 0.117, *T*_max_ = 0.188
5524 measured reflections
2934 independent reflections
2727 reflections with *I* > 2σ(*I*)
*R*_int_ = 0.037

#### Refinement

*R*[*F* ^2^ > 2σ(*F* ^2^)] = 0.026
*wR*(*F* ^2^) = 0.064
*S* = 1.00
2934 reflections
181 parameters
H-atom parameters constrained
Δρ_max_ = 2.04 e Å^−3^
Δρ_min_ = −0.97 e Å^−3^

### 

Data collection: *PROCESS-AUTO* (Rigaku/MSC, 2006 ▶); cell refinement: *PROCESS-AUTO*; data reduction: *CrystalStructure* (Rigaku/MSC, 2006 ▶); program(s) used to solve structure: *DIRDIF99* (Beurskens *et al.*, 1999 ▶); program(s) used to refine structure: *SHELXL97* (Sheldrick, 2008 ▶); molecular graphics: *ORTEP-3* (Farrugia, 1997 ▶); software used to prepare material for publication: *CrystalStructure*.

## Supplementary Material

Crystal structure: contains datablocks I, global. DOI: 10.1107/S1600536810013449/om2328sup1.cif

Structure factors: contains datablocks I. DOI: 10.1107/S1600536810013449/om2328Isup2.hkl

Additional supplementary materials:  crystallographic information; 3D view; checkCIF report

## Figures and Tables

**Table:** 

U1—O1	1.777 (4)
U1—O2	1.784 (4)
U1—O3^i^	2.303 (4)
U1—O3	2.315 (4)
U1—O5	2.428 (4)
U1—O4	2.436 (4)
U1—O6	2.515 (4)
U1—O7	2.523 (4)
O3—O3^i^	1.492 (8)

**Table:** 

O1—U1—O2	175.6 (2)
O3^i^—U1—O3	37.70 (19)

Symmetry code: (i) 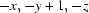.

**Table 2:** Hydrogen-bond geometry (Å, °)

*D*—H⋯*A*	*D*—H	H⋯*A*	*D*⋯*A*	*D*—H⋯*A*
N1—H1⋯O4^ii^	0.88	2.03	2.885 (6)	165
N2—H2⋯O2^iii^	0.88	2.31	3.127 (7)	156

Symmetry codes: (ii) 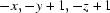; (iii) 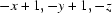.

**Fig. 1.**
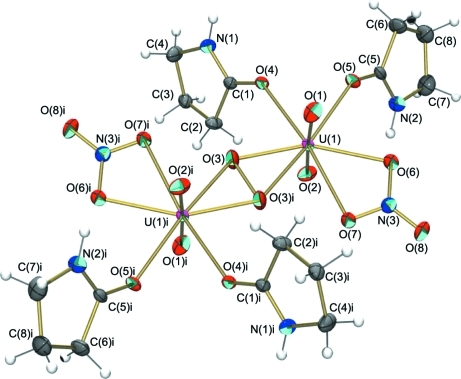
A drawing of µ-η2:η2-peroxo-bis[nitratobis(pyrrolidine-2-one)dioxouranium(VI)] (1) showing 50% probability displacement ellipsoids. Symmetry code: (i) -x, -y+1, -z.

**Fig. 2.**
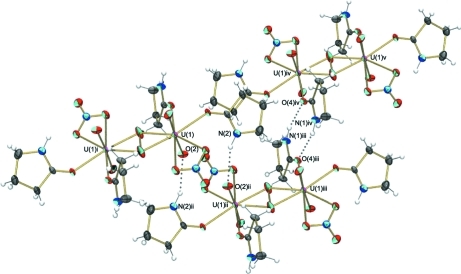
Intermolecular hydrogen bonds. Symmetry codes: (i) -x, -y+1, -z; (ii) -x+1, -y+1, -z; (iii) x+1, y, z; (iv) -x+1, -y+1, -z+1; (v) x+1, y, z+1.

## supplementary crystallographic information

### Comment

The molecular structure of the title compound is shown in Fig. 1. The uranium atom is surrounded by eight O atoms; two are at the axial position, as part of the uranyl cation, and the remaining six O from pyrrolidine-2-ones, nitrates, and peroxo which form a distorted-hexagonal equatorial plane. The peroxide unit shows "side-on" coordination and connects two U, *i.e.*, µ-η^2^:η^2^-O_2_. The bond lengths between U and the axial O are 1.78 Å (mean), indicating that oxidation state of U is exclusively 6+, i.e., UO_2_^2+^ (see related literature; *cf.* 1.84-1.91 Å for U^V^O_2_^+^, Ikeda *et al.*, 2007, Takao *et al.*, 2009). Furthermore, the O—O distance is 1.492 (8) Å, which is typical of peroxide, O_2_^2–^ (Vaska, 1976). One intermolecular hydrogen bonds is found between N—H of pyrrolidine-2-one and the coordinating O of the same ligand in the neighboring complex. A second intermolecular hydrogen bond is found between the N—H of the other pyrrolidine-2-one and one of the uranyl oxo atoms, see Fig. 2.

Photochemically excited *UO_2_^2+^ is a potent and long-lived oxidant for organic and inorganic substrates including the solvent. After the oxidation, UO_2_^+^ is generated as a short-lived intermediate. This species is very unstable and immediately oxidized by dioxygen molecule. As a result, the initial UO_2_^2+^ is regenerated, and the photo-induced catalytic cycle is repeated until termination of photo irradiation or complete conversion of the substrate. This reaction affords peroxide as a by-product. As described in Experimental, compound 1 was unexpectedly obtained from an ethanolic solution dissolving UO_2_(NO_3_)_2_6H_2_O and pyrrolidine-2-one under sunlight. The peroxo ligand most likely arose from oxidative addition of atmospheric dioxygen molecule to the UO_2_^+^ intermediate through the above-mentioned catalytic oxidation of ethanol by the photo-excited *UO_2_^2+^. A similar reaction was speculated in some of the former studies which also described incidental deposition of the uranyl-peroxo complexes [Charpin *et al.* (1985); Doyle *et al.* (1993); John *et al.* (2004)].

### Experimental

Pyrrolidine-2-one (2-pyrr, 0.11 g) was added dropwise into a hot ethanol solution (5 ml) dissolving uranyl(VI) nitrate hexahydrate (0.32 g) with vigorous stirring. After stirring for several minutes, the mixture was cooled to room temperature. Yellow crystals of UO_2_(NO_3_)_2_(2-pyrr)_2_ were removed by filtration. The supernatant was stored under the sunlight. After several days, orange platelet crystals of {[UO_2_NO_3_(C_4_H_7_NO)_2_]_2_O_2_} subsequently deposited, which were suitable for the X-ray diffraction experiment.

### Refinement

All hydrogen atoms were geometrically positioned (C—H 0.99 Å, N—H 0.88 Å) and refined as riding on their parent atoms, with *U*_iso_(H) = 1.2 *U*_eq_(C,*N*).

### Crystal data

**Table:**

[U_2_(NO_3_)_2_O_4_(O_2_)(C_4_H_7_NO)_4_]	*Z* = 1
*M**_r_* = 1036.50	*F*(000) = 478
Triclinic, *P*1	*D*_x_ = 2.650 Mg m^−^^3^
*a* = 8.783 (2) Å	Mo *K*α radiation, λ = 0.71073 Å
*b* = 8.899 (3) Å	Cell parameters from 6275 reflections
*c* = 9.587 (3) Å	θ = 3.1–27.5°
α = 68.24 (3)°	µ = 12.54 mm^−^^1^
β = 81.30 (2)°	*T* = 173 K
γ = 68.96 (2)°	Platelet, orange
*V* = 649.4 (3) Å^3^	0.30 × 0.20 × 0.20 mm

### Data collection

**Table:**

Rigaku R-AXIS RAPID diffractometer	2934 independent reflections
Radiation source: fine-focus sealed tube	2727 reflections with *I* > 2σ(*I*)
graphite	*R*_int_ = 0.037
Detector resolution: 10.00 pixels mm^-1^	θ_max_ = 27.5°, θ_min_ = 3.1°
ω scans	*h* = −11→11
Absorption correction: numerical (*NUMABS*; Higashi, 1999)	*k* = −11→11
*T*_min_ = 0.117, *T*_max_ = 0.188	*l* = −12→12
5524 measured reflections	

### Refinement

**Table:**

Refinement on *F*^2^	Primary atom site location: structure-invariant direct methods
Least-squares matrix: full	Secondary atom site location: difference Fourier map
*R*[*F*^2^ > 2σ(*F*^2^)] = 0.026	Hydrogen site location: inferred from neighbouring sites
*wR*(*F*^2^) = 0.064	H-atom parameters constrained
*S* = 1.00	*w* = 1/[σ^2^(*F*_o_^2^) + (0.0387*P*)^2^ + 2.5299*P*] where *P* = (*F*_o_^2^ + 2*F*_c_^2^)/3
2934 reflections	(Δ/σ)_max_ < 0.001
181 parameters	Δρ_max_ = 2.04 e Å^−^^3^
0 restraints	Δρ_min_ = −0.97 e Å^−^^3^

### Special details

**Table:**

Geometry. All esds (except the esd in the dihedral angle between two l.s. planes) are estimated using the full covariance matrix. The cell esds are taken into account individually in the estimation of esds in distances, angles and torsion angles; correlations between esds in cell parameters are only used when they are defined by crystal symmetry. An approximate (isotropic) treatment of cell esds is used for estimating esds involving l.s. planes.

### Fractional atomic coordinates and isotropic or equivalent isotropic displacement parameters (Å^2^)

**Table:**

	*x*	*y*	*z*	*U*_iso_*/*U*_eq_	
U1	0.21945 (2)	0.38333 (2)	0.134424 (19)	0.01484 (7)	
O1	0.1573 (5)	0.2025 (6)	0.2384 (5)	0.0283 (9)	
O2	0.2956 (6)	0.5569 (5)	0.0387 (5)	0.0290 (9)	
O3	−0.0454 (5)	0.5419 (7)	0.0555 (5)	0.0427 (13)	
O4	0.0681 (4)	0.5317 (5)	0.3068 (4)	0.0184 (7)	
O5	0.3773 (5)	0.2945 (5)	0.3554 (4)	0.0233 (8)	
O6	0.5044 (5)	0.1836 (5)	0.1196 (4)	0.0253 (8)	
O7	0.3597 (5)	0.2765 (6)	−0.0771 (5)	0.0274 (9)	
O8	0.6150 (5)	0.1159 (6)	−0.0778 (5)	0.0320 (10)	
N1	−0.1016 (6)	0.7323 (6)	0.4011 (5)	0.0219 (9)	
H1	−0.1109	0.6587	0.4908	0.026*	
N2	0.6174 (6)	0.3525 (6)	0.3046 (6)	0.0252 (10)	
H2	0.6093	0.3951	0.2062	0.030*	
N3	0.4985 (6)	0.1888 (6)	−0.0142 (5)	0.0220 (9)	
C1	−0.0129 (6)	0.6859 (6)	0.2925 (6)	0.0162 (9)	
C2	−0.0222 (7)	0.8410 (7)	0.1547 (6)	0.0232 (11)	
H2A	−0.0899	0.8478	0.0774	0.028*	
H2B	0.0879	0.8390	0.1114	0.028*	
C3	−0.1017 (7)	0.9916 (7)	0.2126 (6)	0.0220 (11)	
H3A	−0.0184	1.0345	0.2284	0.026*	
H3B	−0.1825	1.0869	0.1408	0.026*	
C4	−0.1845 (8)	0.9175 (7)	0.3610 (7)	0.0289 (13)	
H4A	−0.1688	0.9593	0.4384	0.035*	
H4B	−0.3028	0.9477	0.3484	0.035*	
C5	0.5058 (6)	0.2954 (7)	0.3950 (6)	0.0183 (10)	
C6	0.5538 (7)	0.2302 (9)	0.5552 (7)	0.0285 (12)	
H6A	0.5754	0.1051	0.6004	0.034*	
H6B	0.4671	0.2882	0.6150	0.034*	
C7	0.7558 (8)	0.3392 (9)	0.3818 (8)	0.0336 (14)	
H7A	0.7700	0.4524	0.3536	0.040*	
H7B	0.8580	0.2579	0.3574	0.040*	
C8	0.7105 (8)	0.2736 (9)	0.5478 (7)	0.0314 (13)	
H8A	0.6906	0.3626	0.5933	0.038*	
H8B	0.7993	0.1703	0.6025	0.038*	

### Atomic displacement parameters (Å^2^)

**Table:**

	*U*^11^	*U*^22^	*U*^33^	*U*^12^	*U*^13^	*U*^23^
U1	0.01539 (10)	0.01631 (10)	0.01212 (10)	−0.00364 (7)	−0.00246 (6)	−0.00480 (7)
O1	0.026 (2)	0.029 (2)	0.036 (2)	−0.0151 (17)	0.0063 (17)	−0.0150 (19)
O2	0.039 (2)	0.024 (2)	0.023 (2)	−0.0135 (18)	0.0088 (17)	−0.0083 (17)
O3	0.030 (2)	0.058 (3)	0.033 (2)	0.019 (2)	−0.0158 (19)	−0.035 (3)
O4	0.0237 (18)	0.0151 (17)	0.0135 (16)	−0.0020 (14)	−0.0020 (14)	−0.0053 (14)
O5	0.0199 (18)	0.034 (2)	0.0158 (18)	−0.0079 (16)	−0.0062 (14)	−0.0077 (16)
O6	0.0233 (19)	0.030 (2)	0.0205 (19)	−0.0036 (16)	−0.0038 (15)	−0.0099 (17)
O7	0.026 (2)	0.030 (2)	0.0210 (19)	0.0021 (16)	−0.0052 (16)	−0.0117 (17)
O8	0.024 (2)	0.034 (2)	0.034 (2)	−0.0005 (17)	0.0046 (17)	−0.019 (2)
N1	0.031 (2)	0.012 (2)	0.018 (2)	−0.0049 (17)	0.0034 (18)	−0.0043 (17)
N2	0.028 (2)	0.027 (2)	0.020 (2)	−0.011 (2)	−0.0015 (19)	−0.006 (2)
N3	0.024 (2)	0.022 (2)	0.021 (2)	−0.0057 (18)	−0.0002 (18)	−0.0099 (19)
C1	0.019 (2)	0.016 (2)	0.015 (2)	−0.0066 (18)	−0.0017 (18)	−0.0056 (19)
C2	0.035 (3)	0.017 (2)	0.015 (2)	−0.009 (2)	0.001 (2)	−0.002 (2)
C3	0.022 (2)	0.019 (3)	0.021 (3)	−0.006 (2)	−0.001 (2)	−0.002 (2)
C4	0.036 (3)	0.017 (3)	0.025 (3)	−0.002 (2)	0.007 (2)	−0.006 (2)
C5	0.021 (2)	0.015 (2)	0.015 (2)	−0.0003 (18)	−0.0060 (19)	−0.0050 (19)
C6	0.027 (3)	0.043 (4)	0.020 (3)	−0.015 (3)	−0.005 (2)	−0.011 (3)
C7	0.027 (3)	0.039 (4)	0.042 (4)	−0.016 (3)	0.001 (3)	−0.018 (3)
C8	0.029 (3)	0.041 (4)	0.030 (3)	−0.013 (3)	−0.007 (2)	−0.015 (3)

### Geometric parameters (Å, °)

**Table:**

U1—O1	1.777 (4)	N2—C7	1.464 (8)
U1—O2	1.784 (4)	N2—H2	0.8800
U1—O3^i^	2.303 (4)	C1—C2	1.503 (7)
U1—O3	2.315 (4)	C2—C3	1.534 (8)
U1—O5	2.428 (4)	C2—H2A	0.9900
U1—O4	2.436 (4)	C2—H2B	0.9900
U1—O6	2.515 (4)	C3—C4	1.524 (8)
U1—O7	2.523 (4)	C3—H3A	0.9900
U1—N3	2.960 (5)	C3—H3B	0.9900
O3—O3^i^	1.492 (8)	C4—H4A	0.9900
O3—U1^i^	2.303 (4)	C4—H4B	0.9900
O4—C1	1.264 (6)	C5—C6	1.494 (7)
O5—C5	1.247 (6)	C6—C8	1.543 (8)
O6—N3	1.275 (6)	C6—H6A	0.9900
O7—N3	1.284 (6)	C6—H6B	0.9900
O8—N3	1.211 (6)	C7—C8	1.523 (9)
N1—C1	1.305 (7)	C7—H7A	0.9900
N1—C4	1.465 (7)	C7—H7B	0.9900
N1—H1	0.8800	C8—H8A	0.9900
N2—C5	1.321 (7)	C8—H8B	0.9900
			
O1—U1—O2	175.6 (2)	O8—N3—O6	122.6 (5)
O1—U1—O3^i^	90.6 (2)	O8—N3—O7	122.2 (5)
O2—U1—O3^i^	93.5 (2)	O6—N3—O7	115.2 (4)
O1—U1—O3	90.0 (2)	O8—N3—U1	176.9 (4)
O2—U1—O3	94.2 (2)	O6—N3—U1	57.5 (2)
O3^i^—U1—O3	37.70 (19)	O7—N3—U1	57.9 (2)
O1—U1—O5	83.92 (18)	O4—C1—N1	123.2 (5)
O2—U1—O5	92.05 (18)	O4—C1—C2	126.9 (5)
O3^i^—U1—O5	173.10 (16)	N1—C1—C2	109.9 (4)
O3—U1—O5	137.69 (14)	C1—C2—C3	103.7 (4)
O1—U1—O4	91.03 (16)	C1—C2—H2A	111.0
O2—U1—O4	89.17 (16)	C3—C2—H2A	111.0
O3^i^—U1—O4	106.98 (13)	C1—C2—H2B	111.0
O3—U1—O4	69.31 (13)	C3—C2—H2B	111.0
O5—U1—O4	68.98 (13)	H2A—C2—H2B	109.0
O1—U1—O6	89.08 (17)	C4—C3—C2	104.6 (4)
O2—U1—O6	87.70 (18)	C4—C3—H3A	110.8
O3^i^—U1—O6	117.42 (13)	C2—C3—H3A	110.8
O3—U1—O6	155.09 (14)	C4—C3—H3B	110.8
O5—U1—O6	66.88 (13)	C2—C3—H3B	110.8
O4—U1—O6	135.59 (13)	H3A—C3—H3B	108.9
O1—U1—O7	96.11 (18)	N1—C4—C3	103.3 (4)
O2—U1—O7	84.17 (17)	N1—C4—H4A	111.1
O3^i^—U1—O7	67.09 (14)	C3—C4—H4A	111.1
O3—U1—O7	104.63 (14)	N1—C4—H4B	111.1
O5—U1—O7	117.64 (13)	C3—C4—H4B	111.1
O4—U1—O7	170.69 (12)	H4A—C4—H4B	109.1
O6—U1—O7	50.79 (13)	O5—C5—N2	125.9 (5)
O1—U1—N3	93.83 (17)	O5—C5—C6	123.6 (5)
O2—U1—N3	84.52 (17)	N2—C5—C6	110.5 (5)
O3^i^—U1—N3	92.51 (14)	C5—C6—C8	104.3 (5)
O3—U1—N3	130.14 (14)	C5—C6—H6A	110.9
O5—U1—N3	92.10 (13)	C8—C6—H6A	110.9
O4—U1—N3	159.86 (13)	C5—C6—H6B	110.9
O6—U1—N3	25.29 (13)	C8—C6—H6B	110.9
O7—U1—N3	25.54 (13)	H6A—C6—H6B	108.9
O3^i^—O3—U1^i^	71.6 (3)	N2—C7—C8	104.1 (5)
O3^i^—O3—U1	70.7 (3)	N2—C7—H7A	110.9
U1^i^—O3—U1	142.30 (19)	C8—C7—H7A	110.9
C1—O4—U1	134.6 (3)	N2—C7—H7B	110.9
C5—O5—U1	141.9 (4)	C8—C7—H7B	110.9
N3—O6—U1	97.2 (3)	H7A—C7—H7B	109.0
N3—O7—U1	96.6 (3)	C7—C8—C6	106.1 (5)
C1—N1—C4	114.3 (5)	C7—C8—H8A	110.5
C1—N1—H1	122.9	C6—C8—H8A	110.5
C4—N1—H1	122.9	C7—C8—H8B	110.5
C5—N2—C7	114.4 (5)	C6—C8—H8B	110.5
C5—N2—H2	122.8	H8A—C8—H8B	108.7
C7—N2—H2	122.8		
			
O1—U1—O3—O3^i^	−91.0 (5)	U1—O6—N3—O8	176.3 (5)
O2—U1—O3—O3^i^	90.3 (5)	U1—O6—N3—O7	−3.9 (5)
O5—U1—O3—O3^i^	−172.0 (3)	U1—O7—N3—O8	−176.3 (5)
O4—U1—O3—O3^i^	177.9 (5)	U1—O7—N3—O6	3.9 (5)
O6—U1—O3—O3^i^	−3.2 (8)	O1—U1—N3—O8	−173 (7)
O7—U1—O3—O3^i^	5.3 (5)	O2—U1—N3—O8	3(7)
N3—U1—O3—O3^i^	4.0 (6)	O3^i^—U1—N3—O8	96 (7)
O1—U1—O3—U1^i^	−91.0 (5)	O3—U1—N3—O8	94 (7)
O2—U1—O3—U1^i^	90.3 (5)	O5—U1—N3—O8	−89 (7)
O3^i^—U1—O3—U1^i^	0.000 (2)	O4—U1—N3—O8	−69 (7)
O5—U1—O3—U1^i^	−172.0 (3)	O6—U1—N3—O8	−93 (7)
O4—U1—O3—U1^i^	177.9 (5)	O7—U1—N3—O8	91 (7)
O6—U1—O3—U1^i^	−3.2 (8)	O1—U1—N3—O6	−79.7 (3)
O7—U1—O3—U1^i^	5.3 (5)	O2—U1—N3—O6	96.2 (3)
N3—U1—O3—U1^i^	4.0 (6)	O3^i^—U1—N3—O6	−170.5 (3)
O1—U1—O4—C1	−144.0 (5)	O3—U1—N3—O6	−172.9 (3)
O2—U1—O4—C1	40.4 (5)	O5—U1—N3—O6	4.4 (3)
O3^i^—U1—O4—C1	−53.0 (5)	O4—U1—N3—O6	23.9 (6)
O3—U1—O4—C1	−54.3 (5)	O7—U1—N3—O6	−175.8 (5)
O5—U1—O4—C1	132.9 (5)	O1—U1—N3—O7	96.2 (3)
O6—U1—O4—C1	126.3 (4)	O2—U1—N3—O7	−87.9 (3)
O7—U1—O4—C1	−3.8 (11)	O3^i^—U1—N3—O7	5.3 (4)
N3—U1—O4—C1	111.9 (5)	O3—U1—N3—O7	2.9 (4)
O1—U1—O5—C5	149.6 (6)	O5—U1—N3—O7	−179.8 (3)
O2—U1—O5—C5	−28.6 (6)	O4—U1—N3—O7	−160.2 (3)
O3^i^—U1—O5—C5	−172.1 (11)	O6—U1—N3—O7	175.8 (5)
O3—U1—O5—C5	−127.0 (6)	U1—O4—C1—N1	171.1 (4)
O4—U1—O5—C5	−116.9 (6)	U1—O4—C1—C2	−9.6 (8)
O6—U1—O5—C5	58.0 (6)	C4—N1—C1—O4	179.5 (5)
O7—U1—O5—C5	55.9 (6)	C4—N1—C1—C2	0.2 (7)
N3—U1—O5—C5	56.0 (6)	O4—C1—C2—C3	−166.8 (5)
O1—U1—O6—N3	101.0 (3)	N1—C1—C2—C3	12.5 (6)
O2—U1—O6—N3	−82.0 (3)	C1—C2—C3—C4	−19.4 (6)
O3^i^—U1—O6—N3	10.7 (4)	C1—N1—C4—C3	−12.8 (7)
O3—U1—O6—N3	12.9 (6)	C2—C3—C4—N1	19.4 (6)
O5—U1—O6—N3	−175.2 (3)	U1—O5—C5—N2	−3.6 (9)
O4—U1—O6—N3	−168.5 (3)	U1—O5—C5—C6	176.3 (4)
O7—U1—O6—N3	2.3 (3)	C7—N2—C5—O5	179.5 (5)
O1—U1—O7—N3	−86.1 (3)	C7—N2—C5—C6	−0.4 (7)
O2—U1—O7—N3	89.5 (3)	O5—C5—C6—C8	−174.8 (5)
O3^i^—U1—O7—N3	−174.2 (4)	N2—C5—C6—C8	5.1 (7)
O3—U1—O7—N3	−177.7 (3)	C5—N2—C7—C8	−4.6 (7)
O5—U1—O7—N3	0.2 (4)	N2—C7—C8—C6	7.3 (7)
O4—U1—O7—N3	134.0 (7)	C5—C6—C8—C7	−7.6 (7)
O6—U1—O7—N3	−2.3 (3)		

Symmetry codes: (i) −*x*, −*y*+1, −*z*.

### Hydrogen-bond geometry (Å, °)

**Table:**

*D*—H···*A*	*D*—H	H···*A*	*D*···*A*	*D*—H···*A*
N1—H1···O4^ii^	0.88	2.03	2.885 (6)	165
N2—H2···O2^iii^	0.88	2.31	3.127 (7)	156

Symmetry codes: (ii) −*x*, −*y*+1, −*z*+1; (iii) −*x*+1, −*y*+1, −*z*.
